# AFTGAN: prediction of multi-type PPI based on attention free transformer and graph attention network

**DOI:** 10.1093/bioinformatics/btad052

**Published:** 2023-01-24

**Authors:** Yanlei Kang, Arne Elofsson, Yunliang Jiang, Weihong Huang, Minzhe Yu, Zhong Li

**Affiliations:** Zhejiang Province Key Laboratory of Smart Management & Application of Modern Agricultural Resources, School of Information Engineering, Huzhou University, Huzhou, Zhejiang 313000, China; Department of Biochemistry and Biophysics, Science for Life Laboratory, Stockholm University, Stockholm, Solna 17121, Sweden; School of Computer Science and Technology, Zhejiang Normal University, Jinhua, Zhejiang 321004, China; College of Science, Zhejiang Sci-Tech University, Hangzhou, Zhejiang 310018, China; College of Science, Zhejiang Sci-Tech University, Hangzhou, Zhejiang 310018, China; Zhejiang Province Key Laboratory of Smart Management & Application of Modern Agricultural Resources, School of Information Engineering, Huzhou University, Huzhou, Zhejiang 313000, China; Department of Biochemistry and Biophysics, Science for Life Laboratory, Stockholm University, Stockholm, Solna 17121, Sweden; College of Science, Zhejiang Sci-Tech University, Hangzhou, Zhejiang 310018, China

## Abstract

**Motivation:**

Protein–protein interaction (PPI) networks and transcriptional regulatory networks are critical in regulating cells and their signaling. A thorough understanding of PPIs can provide more insights into cellular physiology at normal and disease states. Although numerous methods have been proposed to predict PPIs, it is still challenging for interaction prediction between unknown proteins. In this study, a novel neural network named AFTGAN was constructed to predict multi-type PPIs. Regarding feature input, ESM-1b embedding containing much biological information for proteins was added as a protein sequence feature besides amino acid co-occurrence similarity and one-hot coding. An ensemble network was also constructed based on a transformer encoder containing an AFT module (performing the weight operation on vital protein sequence feature information) and graph attention network (extracting the relational features of protein pairs) for the part of the network framework.

**Results:**

The experimental results showed that the Micro-F1 of the AFTGAN based on three partitioning schemes (BFS, DFS and the random mode) on the SHS27K and SHS148K datasets was 0.685, 0.711 and 0.867, as well as 0.745, 0.819 and 0.920, respectively, all higher than that of other popular methods. In addition, the experimental comparisons confirmed the performance superiority of the proposed model for predicting PPIs of unknown proteins on the STRING dataset.

**Availability and implementation:**

The source code is publicly available at https://github.com/1075793472/AFTGAN.

**Supplementary information:**

Supplementary data are available at *Bioinformatics* online.

## 1 Introduction

Typically, individual proteins do their tasks through direct interactions with other proteins, instead of performing any of their functions alone. Accordingly, studying protein–protein interaction (PPI) networks has become a powerful tool for identifying the functional consequence of genetic variation. Many experimental methods detect PPI (Fields and Song, 1989). However, experiment-based methods are expensive and time-consuming, and even if a single experiment detects a PPI, its type cannot be completely judged (Rivas and Fontanillo, 2010). Notably, reliable computational methods learned from accumulated PPI data are urgently required to predict unknown PPIs accurately.

Since it has been confirmed that the amino acid sequence contains all protein information, and it is easy to obtain (Anfinsen, 1972), sequence-based methods have long been applied to model protein associated tasks (e.g. PPI prediction and classification). Early research has been based on machine learning (ML). Guo *et al.* (2008) combined auto covariance (AC) and support vector machine (SVM) to extract features for predicting the PPI data of *Saccharomyces cerevisiae*. Subsequently, Silberberg *et al.* (2014) proposed the method based on the logistic regression to predict the PPI type. Then, Wong *et al.* (2015) developed a sequence-based method to predict the PPI data of yeast by combining the physicochemical property matrix of proteins with the rotation forest algorithm. The above methods all provide feasible solutions, whereas their performance is limited by the inability to effectively extract PPI features and the expressiveness limitation of prediction models.

Deep learning (DL) has extensively been used in various bioinformatics problems for its powerful model expression capability. Li *et al.* (2018) proposed a deep neural network framework (DNN-PPI) to predict PPIs from features automatically learned from protein-level sequences. Two interacting protein sequences in this model are sequentially input into encoding, embedding, convolution neural network (CNN) (Gu *et al.*, 2018) and long short-term memory (LSTM) network (Hochreiter and Schmidhuber, 1997). Subsequently, the connection vector of two outputs is concatenated as the input of the fully connected neural network for the learning. Moreover, Hashemifar *et al.* (2018) proposed a DPPI model to predict PPIs only based on sequence information. This DPPT model can effectively apply a deep, Siamese-like convolutional neural network combined with random projection and data augmentation to predict PPI by leveraging the high-quality experimental PPI data and the evolutionary information of the predicted protein pairs. Chen *et al.* (2019) introduced an end-to-end framework (PIPR) for PPI prediction through protein sequences. This PIPR uses strong local features and contextual information by incorporating a deep residual recurrent convolutional neural network in the Siamese architecture, which is beneficial to extract the interaction features of protein sequences.

There have been many advances in the feature representation of proteins over the past few years. Dutta and Saha (2020) proposed a novel DL architecture to extract multimodal information from existing protein structure and textual information in biomedical literature. Nambiar *et al.* (2020) provided a neural network based on Transformer (Vaswani *et al.*, 2017) to generate protein pre-training embedding. This model was fine-tuned to cope with two different protein prediction tasks (protein family classification and PPI prediction). Rives *et al.* (2021) used unsupervised learning to train a deep contextual language model (ESM-1b) on 86 billion amino acids across 250 million protein sequences spanning evolutionary diversity for the feature representation of protein sequences by Transformers. The resulting model contains information of biological properties in its representation, which is learned from sequence data alone. The learned representation space shows a multiscale organization reflecting structure from the level of biochemical properties of amino acids to remote homology of proteins. Information about secondary and tertiary structure is also encoded in the representations and can be identified by linear projections. Representation learning from the sequence produces features that can be used for a variety of prediction tasks without prior knowledge of evolution and structure. Next, Meier *et al.* (2021) used the Uniref90_2020-03 dataset (Suzek *et al.*, 2007) and trained ESM-1v, a 650M parameter Transformer language model on 98 million different protein sequences, using the ESM-1b framework and masked language modeling approached by Rives *et al.* This model was also learned on sequences without any supervision of functional experimental measurements.

Yang *et al.* (2020) considered the structural information of PPI network for the prediction (e.g. their degrees, positions and neighboring nodes in the graph). On that basis, they introduced an optimized graph representation learning method, which can predict PPIs based on the sequence information and graph structure. Furthermore, they used a representation learning model and employed a graph-based DL method (Kipf and Welling, 2017) for PPI prediction. However, their method cannot be extended to the multi-label PPI classification. Lv *et al.* (2021) considered the interactions between unknown proteins to solve this problem from evaluation and methodological aspects. They designed a novel evaluation framework that fully considers interactions between unknown proteins and provided consistent evaluations across datasets. Besides, they suggested that the correlation between proteins should provide useful information to analyze the PPI of unknown proteins. Subsequently, they proposed a graph neural network-based method (GNN-PPI) to predict new interactions between proteins. However, since the protein sequence feature input of this method is relatively simple, the network for extracting sequence features is limited and the rich biological information of protein sequence is not fully captured.

To more effectively extract protein information for predicting multi-type PPIs, two attention network frameworks were integrated to construct a learning network as AFTGAN. The main work of this study is presented as follows: (i) The ESM-1b encoding was introduced as the feature input of protein sequence. ESM-1b encoding contains considerable biological information of proteins and can fully express the sequence features of proteins. (ii) The protein sequence feature information was extracted through the Transformer framework including the attention free transformer (AFT) module (Zhai *et al.*, 2021). The AFT module reduces the extra execution of attention operations with time and space complexity by replacing the self-attention part of the dot product, while maintaining excellent efficiency in feature extraction. (iii) The relational features between proteins were extracted through the graph attention network (Veličković et al., 2017). This study can assign different weights to the relationship of different protein nodes and better extract the relationship between proteins through the attention mechanism.

## 2 Datasets, materials and methods

### 2.1 Dataset

Multi-type PPI data (*Homo sapiens*, Taxon ID: 9606) from the STRING dataset (Szklarczyk *et al.*, 2019) were used to evaluate the proposed model. The STRING dataset collects, evaluates and integrates PPI information and builds a comprehensive and objective PPI network, including directly linked (physical) and indirectly linked (functional) interactions. The STRING dataset classifies PPIs into seven types: reaction, binding, post-translational modifications (ptmod), activation, inhibition, catalysis and expression. The respective set of interacting protein pairs contains at least one of the above interactions. Referred to Chen *et al.*’s method (Chen *et al.*, 2019) which randomly selected 1690 and 5189 proteins from the *H.sapiens* subset of the STRING, the proposed AFTGAN was compared to other methods under the same dataset condition. As a result, <40% sequence identity was shared, and two subsets, SHS27K and SHS148K, were generated, containing 7624 and 44 488 multi-type PPIs, respectively. Besides, a new sub-dataset from SHS27K with <20% sequence identity was produced for the PPI prediction test and comparison. Moreover, all PPIs of *H.sapiens* were used as our test dataset (tSTRING). It contains 15 335 proteins and 593 397 PPIs, which were applied to test the performance of the PPI prediction model on the unknown proteins. The above PPI datasets with different size and sequence identity were used to evaluate the proposed model and other methods in Section 3.

For above datasets, breadth-first search (BFS), depth-first search (DFS) and random schemes were employed in the PPI network to construct a test set for evaluation (Lv *et al.*, 2021). Proteins closely interact and aggregate in the PPI network based on the BFS partitioning method. Proteins are sparsely distributed in the PPI network and have little interaction with each other based on the DFS partitioning method. Proteins interact with each other in a random manner based on the random mode.

### 2.2 Feature extraction

The protein sequence features used in the proposed model include amino acid co-occurrence similarity encoding, one-hot encoding and ESM-1b embedding feature. Each amino acid *a* is represented as a feature vector $E\left(a\right)=[E_{1}\left(a\right),E_{2}\left(a\right),E_{3}(a)]$, where $E_{1}\left(a\right)$ represents the amino acid co-occurrence similarity with a 5D vector, which can be obtained by pre-training the Skip-Gram model for protein sequences (Mikolov *et al.*, 2013). $E_{2}\left(a\right)$ represents a one-hot encoding of a classification based on the definition of electrostatic and hydrophobic similarity between amino acids, in which 20 natural amino acids are divided into seven types (Shen *et al.*, 2007), as listed in Supplementary Table S1. Selenocysteine, Pyrrolysine and unknown amino acids are classified as the eighth type. $E_{3}\left(a\right)$ denotes a Transformer-based pretrained protein-encoding (ESM-1b embedding) proposed by Rives *et al.* (2021). It used the Masked Language Model (MLM) to pre-train the model. This model employs sequences in the Uniparc database (Consortium, 2010), one of the largest existing sample sets of protein sequences with wide evolutionary diversity. The resulting pre-trained model maps raw sequences to biological feature representations without labels or prior knowledge. ESM-1b encodes the respective protein into a feature of L×1280, where L denotes the protein length. Thus, each amino acid is expressed as $E\left(a\right)\in R^{5+\left(7+1\right)+1280=1293}$.

### 2.3 AFTGAN model

**Fig. 1.**
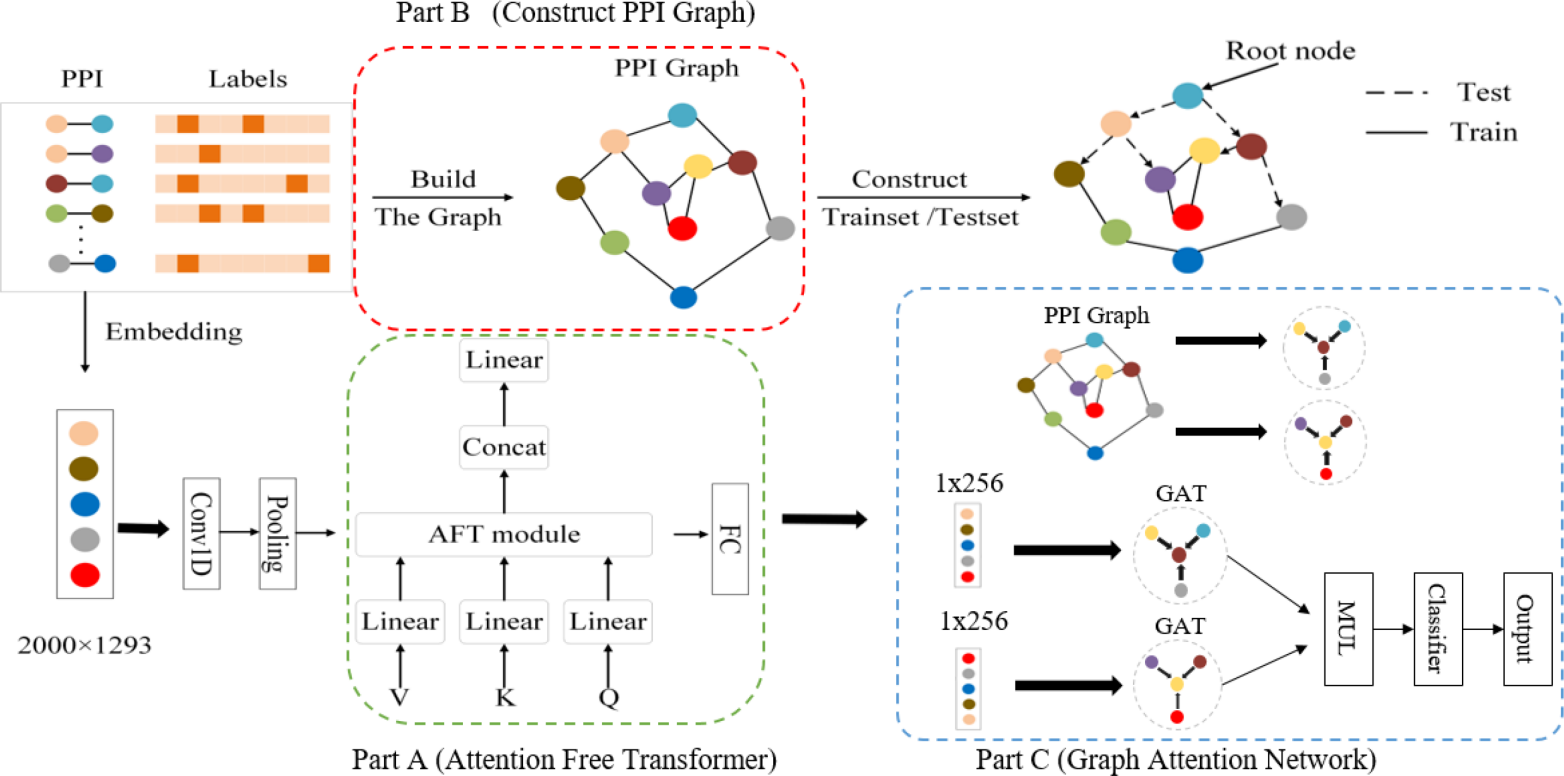
AFTGAN framework structure. The main process consists of three parts: Part A extracts protein sequence information through Attention Free Transformer. Part B constructs a PPI graph through PPI information. PPI graph is not only used to construct the training and test sets, but also used as the input of adjacency matrix of Graph Attention Network. Part C extracts the features between protein pairs through Graph Attention Network

The proposed AFTGAN model mainly comprises three parts as follows. The first part extracts the protein sequence features through 1D convolution and Transformer encoder, including the AFT module. The second part is used to extract the relational features of protein pairs through the graph attention network. The third part performs the multi-label PPI prediction on the extracted features via a fully connected layer. The specific framework is illustrated in Figure 1.

#### 2.3.1 Protein sequence feature extraction

In the proposed AFTGAN, the length of each protein sequence was fixed with 2000 to ensure that the output vector size is fixed. For protein sequences with <2000, the length of 2000 was achieved by supplementing 0 to the end of the input protein sequence. For protein sequences longer than 2000, the sequence was trimmed with the first 2000 amino acid as input. Afterwards, an initial vector of protein sequence is represented with a dimension of 2000×1293.

Subsequently the initial vector was fed into a 1D convolutional layer in the proposed AFTGAN. The 1D convolutional layer has a kernel size of 3 and a padding of 0. This process results in a 1×1998 feature map. Afterward, a 1×666 feature map was obtained through a max pooling layer with a stride length of 3. Next, the extracted features were fed into the Transformer encoder, including the AFT module. To be specific, *Q, V* and *K* are vectors obtained through the linear layer linear(666,666). *Q* represents the query vector, *V* expresses the vector of the queried information, and *K* denotes the vector of the correlation between the queried information and other information. The attention mechanism of the AFT module is expressed as

(1) $${\mathrm{AFT}}_{t,c}=R_{t,c}\cdot \frac{\sum_{u\leq t}W_{t,u}\exp\left(K_{u,c}\right)V_{u,c}}{\sum_{u\leq t}W_{t,u}\exp\left(K_{u,c}\right)},$$

where $R_{t,c}$ is the willingness at the current *t* position to receive information in the c channel; $V_{u,c}$ represents the content of information sent at the u position; $W_{t,u}$ expresses the association strength of *t* and *u* in the channel. The feature map with a dimension of 1×666 is regulated by self-attention through the Transformer, including the AFT module to obtain the semantic features of the protein sequence fully. Lastly, a 1×256 protein sequence feature map is obtained via the fully connected layer.

#### 2.3.2 Relational feature extraction of protein pairs

In the proposed AFTGAN, a PPI graph is defined as $G=\left(P,X\right)$, where nodes are proteins, and edges represent the interactions between proteins. Since the STRING dataset classifies PPI interactions into seven types, seven kinds of PPI graphs were constructed and then input into the network for training. To learn relational features between protein pairs, three graph attention modules (GATs) were stacked.

The initial input of the graph attention layer is a set of node features: $h^{\left(0\right)}=\{{\vec{h}}_{1}^{\left(0\right)},\;{\vec{h}}_{2}^{\left(0\right)},{\vec{h}}_{3}^{\left(0\right)},\ldots ,{\vec{h}}_{m}^{\left(0\right)}\in R^{d_{0}}\}$, where $d_{0}$ = 256, the above features originate from the protein sequence features extracted in Section 2.3.1. After the processing of the i layer of GAT, it captures new node features: $h^{\left(i\right)}=\{{\vec{h}}_{1}^{\left(i\right)},{\vec{h}}_{2}^{\left(i\right)},{\vec{h}}_{3}^{\left(i\right)},\ldots ,{\vec{h}}_{m}^{\left(i\right)}\in R^{d_{i}}\}$. The output node features ${\vec{h}}_{m}^{\left(i\right)}$ are connected by the *K*-head attention

(2) $${\vec{h}}_{m}^{'}=\begin{array}{c} K\\ \left|\right|\\ k=1 \end{array}\sigma (\sum_{n\in N_{m}}a_{mn}^{k}W^{k}{\vec{h}}_{n}^{\prime }),$$

where $W\in R^{d^{\left(i-1\right)}{\times d}^{\left(i\right)}}$ denotes a weighting matrix, which is used for the linear transformation from $h^{\left(i-1\right)}$ to $h^{\left(i\right)}$. $N_{m}$ expresses the set of first-order neighborhoods of node *m*. || represents a join operation. $a_{mn}^{k}$ denotes the normalized attention coefficient computed by the *k*th attention mechanism

(3) $$a_{mn}=\frac{\exp(\mathrm{LeakyReLU}({\vec{a}}^{T}[W{\vec{h}}_{m}||W{\vec{h}}_{n}]))}{\sum_{r\in N_{m}}\exp(\mathrm{LeakyReLU}({\vec{a}}^{T}[W{\vec{h}}_{m}||W{\vec{h}}_{r}]))}$$

GAT uses the self-attention to set the importance to edges between nodes and helps the model learn the structural information of graph. In the proposed model, *K* was set to 2 for the graph attention layers in the first and second layers. Due to the memory size limitation, *K* in GAT of the last layer was set to 1. The final output feature map size was 1024×512.

#### 2.3.3 Multi-label PPI prediction

The protein features PPI$x_{ij}$ learned at the previous stage were used. On that basis, the above features of $p_{i}$ and $p_{j}$ were combined through a dot product operation, and then a fully connected layer (FC) was applied as a classifier for multi-label PPI prediction, which is expressed as ${\hat{y}}_{ij}=\mathrm{FC}(g_{p_{i}}.g_{p_{j}})$. The sequence feature extraction module of proteins and the relational feature extraction module of protein pairs were trained in an end-to-end manner. The multi-task binary cross-entropy was used as the loss function based on a training set $X_{\mathrm{train}}$ and its ground-truth interaction labels $Y_{\mathrm{train}}$

(4) $$L=\sum_{k=0}^{n}\left(\sum_{x_{ij{\in X}_{\mathrm{train}}}}-y_{ij}^{k}l\mathrm{og}{\hat{y}}_{ij}^{k}-(1-y_{ij}^{k})\log(1-{\hat{y}}_{ij}^{k})\right).$$

Unlike other multi-type PPI prediction algorithms considering the features only from the protein sequence or the single graph network, this method learns the features of interrelationships with neighboring nodes from seven types of PPI graphs based on the graph attention mechanism. Accordingly, for the test set $X_{\mathrm{test}}$ constructed by BFS and DFS, the AFTGAN is also capable of generating suitable feature representations for multi-type PPI prediction based on its neighboring nodes. Besides, even if the PPI network $G^{\prime }=(P_{v},X_{\mathrm{train}})$ used in the training process is built only with *X*_train, it can also achieve satisfactory predictions for unknown PPIs $x_{ij}\in X_{\mathrm{test}}$.

### 2.4 AFTGAN model setting

The AFTGAN model primarily includes a protein sequence feature extraction module that comprises a 1D convolution and an encoder of a Transformer, including AFT module, as well as a protein pair relational feature extraction module with a graph attention network stacked by three layers of graph attention layers.

The protein sequence feature extraction module comprises the convolution kernel of 1D convolution set to 3×3 and the padding set to 0. The normalization method is batch norm. The pooling method is Max pooling, and the pool size is set to 3. The d_mode of the AFT module is 666, and *n* is 1. The activation function used by GAT is RELU in the relational feature extraction module of protein pairs (Agarap, 2018). The optimization algorithm in the prediction model is Adam (Kingma and Ba, 2014), with the initial learning rate of 0.001 and the weight_decay set to 5e−4. The learning rate is changed using ReduceLROnPlateau, with the mode set to min, the factor parameter set to 0.5, and the patience set to 20. The dropout is set to 0.5, the batch size is 1024, and a total of 2000 epochs are run.

## 3 Experimental result and discussion

### 3.1 Experimental data and evaluation indicators

In the experiment, BFS, DFS and random partition schemes were used to divide the training and test sets from the PPI dataset, in which the test set accounts for 20%. BFS and DFS schemes achieve completely different results based on different root node settings. The protein sequence as the root node interacts only with a few protein sequences in the experiment, so the root degree threshold was set to *t *=* *5. Moreover, the experimental results were repeated under three random seeds to eliminate the effect of the randomness of data partitioning for the performance of the PPI prediction. The final result was chosen by the average of three experiments and kept to 3 decimal places by the rounding method. The SHS27K and SHS148K datasets were employed for training and testing, respectively, due to the large size of the STRING dataset. All PPIs of *H.sapiens* in STRING (tSTRING) was adopted to evaluate the performance of the model for predicting multi-type PPIs of unknown proteins. We applied four different metrics to evaluate the performance of the model, namely Hamming Loss, Precision, Recall and Micro-F1.

Hamming Loss: It indicates the proportion of wrong samples in all labels, so the smaller the value, the stronger the classification ability of the network.

(5) $$\mathrm{Hamming}\;\mathrm{Loss}=\;\frac{1}{\left|D\right|}\sum_{i=1}^{\left|D\right|}\frac{\mathrm{xor}(x_{i},y_{i})}{\left|L\right|},$$

where |D| represents the total number of samples; |L| denotes the total number of labels; $x_{i}$ and $y_{i}$ represent the prediction result and the ground truth, respectively; xor is the exclusive OR operation.

Precision: It is the ratio of the number of PPI samples classified as having a PPI relationship to the number of samples determined by the classifier as having a PPI relationship.

(6) $$\mathrm{Precision}=\;\frac{\mathrm{TP}}{\mathrm{TP}\;+\;\mathrm{FP}}.$$

Recall: It is the ratio of the number of PPI samples classified as having a PPI relationship to the number of PPI samples.

(7) $$\mathrm{Recall}=\;\frac{\mathrm{TP}}{\mathrm{TP}\;+\;\mathrm{FN}}.$$

Micro-F1: F1 is mainly used for the binary classification, while Macro-F1 and Micro-F1 are used for the multi-label classification. In particular, Micro-F1 is suitable for situations where the data distribution is unbalanced. Since the different PPI types in the dataset we used are unbalanced, Micro-F1 is used as the evaluation metric to evaluate the multi-label PPI prediction performance. It first calculates the total Precision and Recall of all types and then calculates the harmonic mean of Precision and Recall.

(8) $${\mathrm{Precision}}_{\mathrm{Micro}}\;=\;\frac{\sum_{i=1}^{n}{\mathrm{TP}}_{i}}{\sum_{i=1}^{n}{\mathrm{TP}}_{i}+\sum_{i=1}^{n}{\mathrm{FP}}_{i}}.$$

(9) $${\mathrm{Recall}}_{\mathrm{Micro}}=\;\frac{\sum_{i=1}^{n}{\mathrm{TP}}_{i}}{\sum_{i=1}^{n}{\mathrm{TP}}_{i}+\sum_{i=1}^{n}{\mathrm{FN}}_{i}}$$

(10) $$\mathrm{Micro}-F1=\;2\frac{{\mathrm{Precision}}_{\mathrm{micro}}\cdot {\mathrm{Recall}}_{\mathrm{micro}}}{{\mathrm{Precision}}_{\mathrm{micro}}+{\mathrm{Recall}}_{\mathrm{micro}}}$$

where TP, FN, FP and TN represent the results of four types of predictions. True positives (TP) represent actual PPI samples predicted to have a PPI relationship. False negatives (FN) represent actual PPI samples, whereas they are incorrectly predicted as samples that do not have a PPI relationship; false positives (FP) refer to samples that do not have PPI relationships, whereas they are incorrectly predicted to be PPI samples. True negatives (TN) represent correctly predicted samples without PPI relationships.

### 3.2 Comparison with different feature input combinations

For input features, unlike previous methods of predicting PPIs, ESM-1b encoding was added as the input for protein sequence features. To verify whether the input ESM-1b encoding can enhance the performance of multi-type PPI prediction, we tested it on the SHS27K dataset by the ablation experiment. The division results of SHS27K divided by different partitioning schemes are listed in Supplementary Table S2, where the vision node represents a visible protein (appeared in the training dataset) and the invisible node denotes an invisible protein (not appeared in the training dataset), For the test set X, X_BS represents that both interacting proteins are visible proteins, X_ES represents that one of two interacting proteins is a visible protein, and the other is an invisible protein. X_NS suggests that both interacting proteins are invisible proteins. For three partition modes, since the random mode include more visible proteins than BFS and DFS modes, its PPI prediction accuracy is relatively higher than other two modes (Lv *et al.*, 2021).

**Fig. 2.**
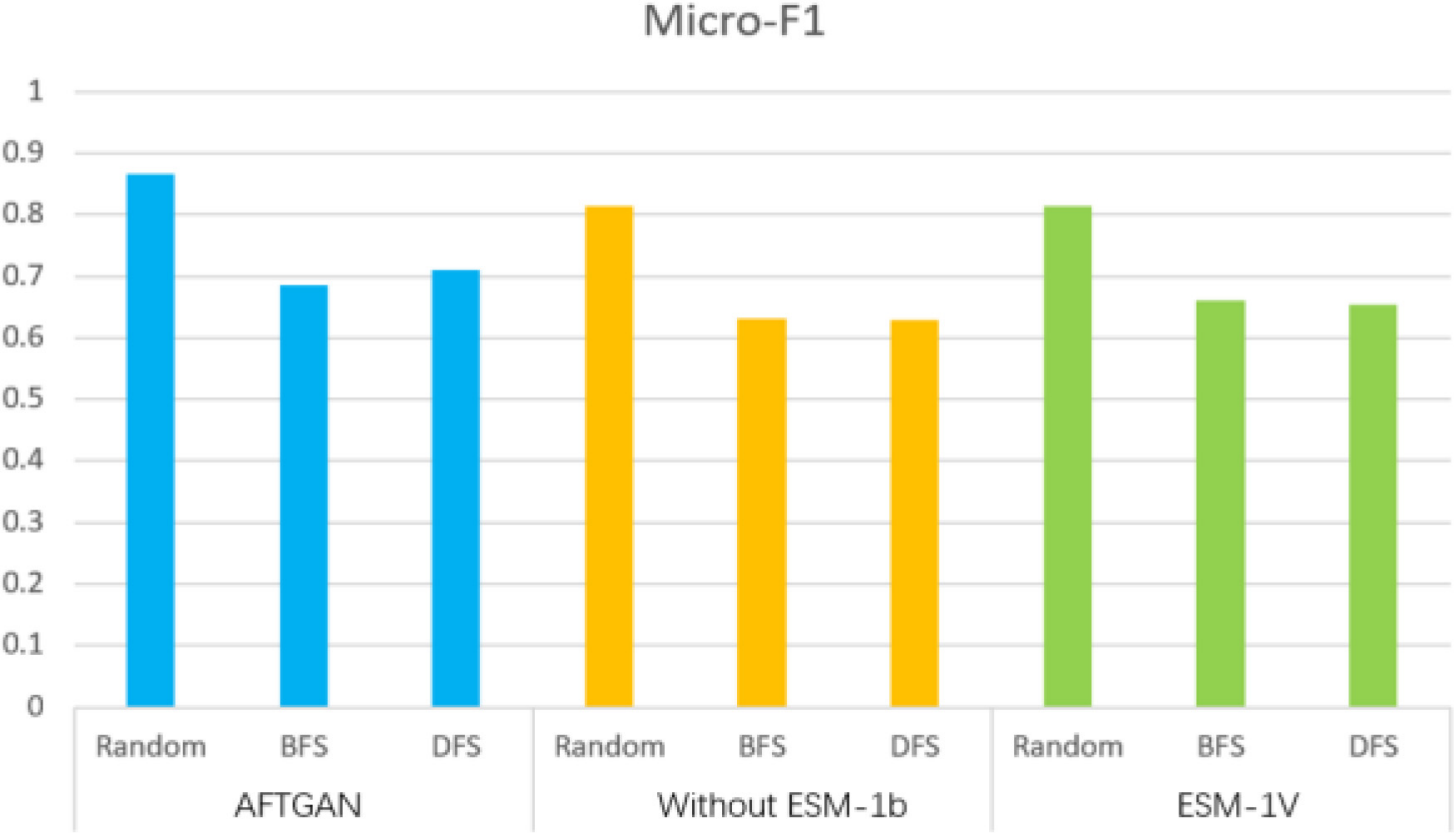
Micro-F1 comparison of different feature combinations on SHS27K

Besides comparing models without the addition of ESM-1b encoding, we also added the recently proposed protein pre-trained encoding ESM-1v encoding (Meier *et al.*, 2021). The detailed Micro-F1 comparison is presented in Figure 2, and the comparisons with other evaluation indicators are listed in Supplementary Table S3. When the partitioning mode was random, the proposed model achieved the Micro-F1 and the Hamming Loss of 0.867 and 0.087, 5.2% and 0.03 higher than the second best-performing feature input models, respectively. When the partitioning mode was BFS, the proposed model achieved the Micro-F1 and the Hamming Loss of 0.685 and 0.191, 2.5% and 0.015 higher than the second best-performing feature input models. When the partitioning mode was DFS, the proposed model achieved the Micro-F1 and the Hamming Loss of 0.711 and 0.185, 5.6% and 0.068 higher than the second best-performing feature input models, respectively. The above results show that the proposed model adding ESM-1b encoding outperforms other two feature combinations in the respective index, especially based on the DFS partitioning mode.

### 3.3 Comparison with different neural network modules

The effects of different network modules on the feature extraction of protein sequences and the relational feature of protein pairs were compared through the ablation experiment. Three experiments were performed on each of three partitioning schemes on the SHS27K dataset, and the average of three experiments was selected as the final result in the respective partitioning scheme. The division results of SHS27K by different partitioning schemes are listed in Supplementary Table S2, and the detailed prediction results with different network modules are listed in Supplementary Table S4.

**Fig. 3.**
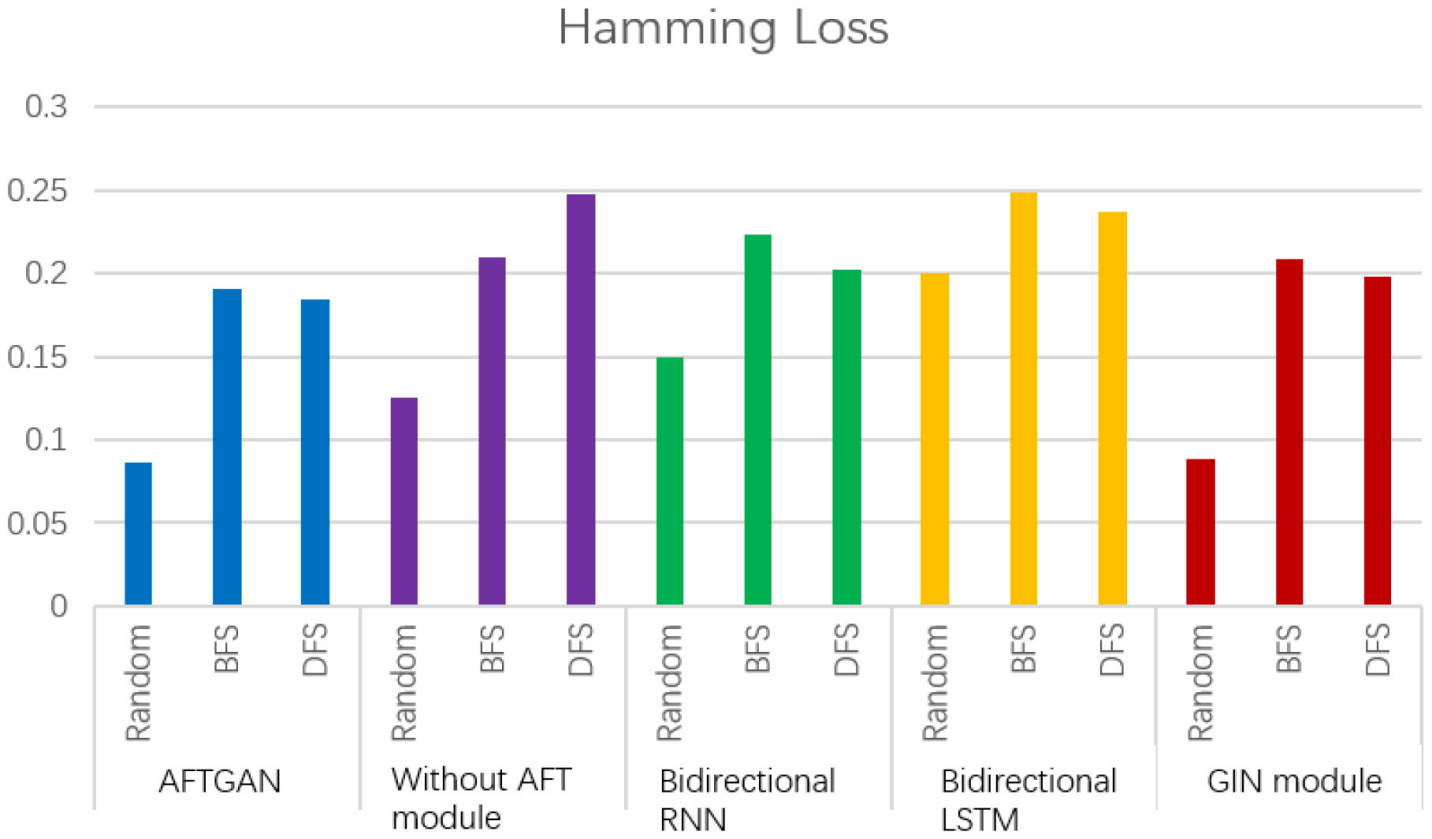
Hamming Loss comparison of different network combinations on SHS27K

To verify the superiority of the AFT module for protein sequence feature extraction, we compared three neural network frameworks: (i) The proposed model without adding AFT module, (ii) The proposed model replacing AFT module by a bidirectional RNN module and (iii) The proposed model replacing AFT module by bidirectional LSTM module. Among them, bidirectional RNN and bidirectional LSTM networks were often used for the feature extraction of protein sequences. Under the random partitioning mode, the proposed model achieved 0.867 and 0.087 in Micro-F1 and Hamming Loss, respectively, outperforming other three modules. Under the BFS partitioning mode, the proposed model achieved 0.685 and 0.191 in Micro-F1 and Hamming Loss, respectively, which are 2.6% and 0.019 higher than the best-performing module in other three neural network frameworks, respectively. Based on the DFS partitioning mode, the proposed model achieved the Micro-F1 and the Hamming Loss of 0.711 and 0.185, 0.3% and 0.013 higher than those of the second best-performing module, respectively. The detailed Hamming Loss comparisons are also shown in Figure 3, which shows that the AFT module can effectively extract the feature from the protein sequences.

Besides, to verify whether the GAT module can effectively extract the relational features of protein pairs, it was replaced by the GIN network (Lv *et al.*, 2021) as a comparison. GIN is a variant of the GNN network, which achieves the satisfactory prediction results by changing the aggregation and updating operations in the GNN network. The proposed model is slightly better than the network with the GIN module based on the random partition mode, suggesting that the graph convolutional network is capable of extracting the relationship features of protein pairs. The proposed model achieved the Micro-F1 and Hamming Loss indicators which are 4%, 0.018 and 0.7%, 0.013 higher than those of the GIN module, respectively, based on the BFS and DFS partition modes. The above results reveal that when there are many unknown proteins, the proposed model outperforms the GIN module in extracting relational features of protein pairs.

### 3.4 Comparison of different methods for multi-type PPI prediction

To verify the performance of the proposed model, the proposed AFTGAN model was compared with three ML models [including SVM (Schuldt *et al.*, 2004), RF (Rodriguez *et al.*, 2006) and LR (Kleinbaum and Klein, 2002)] and four DL models [including DNN-PPI (Li *et al.*, 2018), DPPI (Hashemifar *et al.*, 2018), PIPR (Chen *et al.*, 2019) and GNN-PPI (Lv *et al.*, 2021)] on the SHS27K and SHS148K datasets in predicting multi-type PPIs. Supplementary Tables S2 and S5 list the division results of the test sets (SHS27K and SHS148K) based on different partitioning schemes, respectively. The input features of the three ML methods are common protein features, including AC (Guo *et al.*, 2008) and composition, transition and distribution (Du *et al.*, 2017). The input features of four DL-based methods include amino acid co-occurrence similarity encoding and one-hot encoding of electrostatic and hydrophobic similarity between amino acids.

Table 1 shows the Micro-F1 comparison of different methods on two datasets. As depicted in this table, the above eight models maintain good predicting performance based on the random partition mode. However, only the proposed AFTGAN and GNN-PPI still maintain stable results based on the partition modes of BFS and DFS, while the performance of other methods is reduced significantly. This shows that for unknown proteins, the graph convolution method can still learn the features of adjacent nodes to obtain the relationship features of protein pairs. In addition, the performance of eight models under the DFS division mode is generally higher than that under the BFS division mode, suggesting that the discrete distribution form in the PPI network is relatively easy to learn for unknown proteins.

**Table 1.** Micro-F1 comparison of different methods on SHS27K and SHS148K

Dataset	Partition scheme	Method							
		SVM	RF	LR	DPPI	DNN-PPI	PIPR	GNN-PPI	AFTGAN
SHS27K	Random	0.743	0.785	0.716	0.768	0.802	0.826	0.864	0.867
	BFS	0.436	0.374	0.435	0.478	0.529	0.477	0.620	0.685
	DFS	0.554	0.367	0.484	0.467	0.483	0.537	0.652	0.711
SHS148K	Random	0.803	0.821	0.663	0.784	0.882	0.905	0.918	0.920
	BFS	0.526	0.392	0.481	0.553	0.623	0.653	0.739	0.745
	DFS	0.583	0.446	0.527	0.532	0.587	0.663	0.780	0.819

The comparison results of different prediction methods suggest that the methods based on DL outperform those based on ML because of the powerful expression capability of neural networks for feature extraction. The proposed AFTGAN achieves the best results on all three partition modes on both datasets. Under the random partition mode, the proposed model has stronger learning ability than other models, resulting in satisfactory performance on small datasets. However, the performance advantage of the proposed model decreases with the increase of the data size. The main advantage of the AFTGAN model is that when the dataset is divided into BFS and DFS, the effect of the proposed model is far better than other methods, especially on the small sample dataset.

**Fig. 4.**
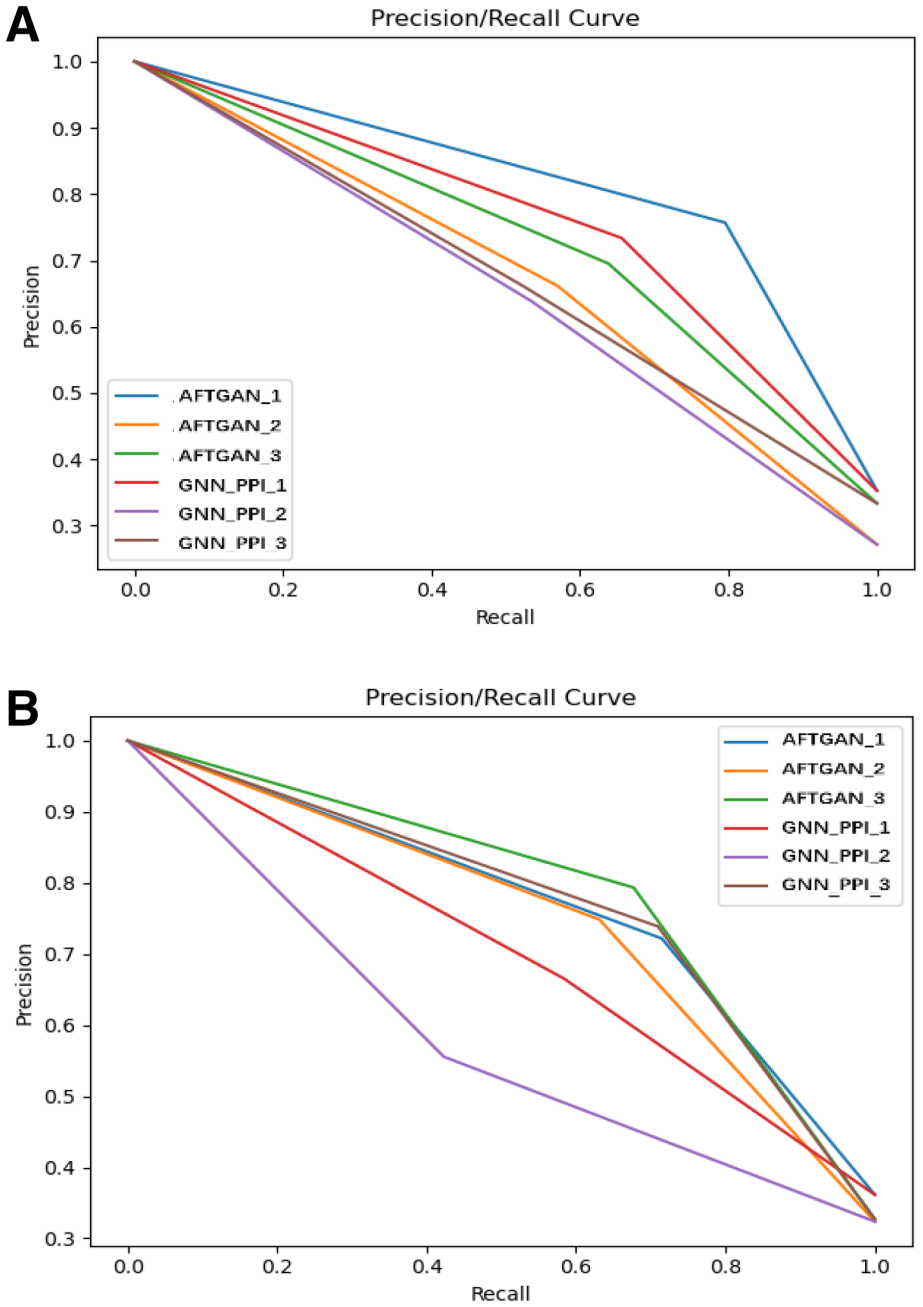
PR curves of three experiments by AFTGAN and GNN_PPI on SHS27K. (**A**) Under the BFS partitioning. (**B**) Under the DFS partitioning

**Fig. 5.**
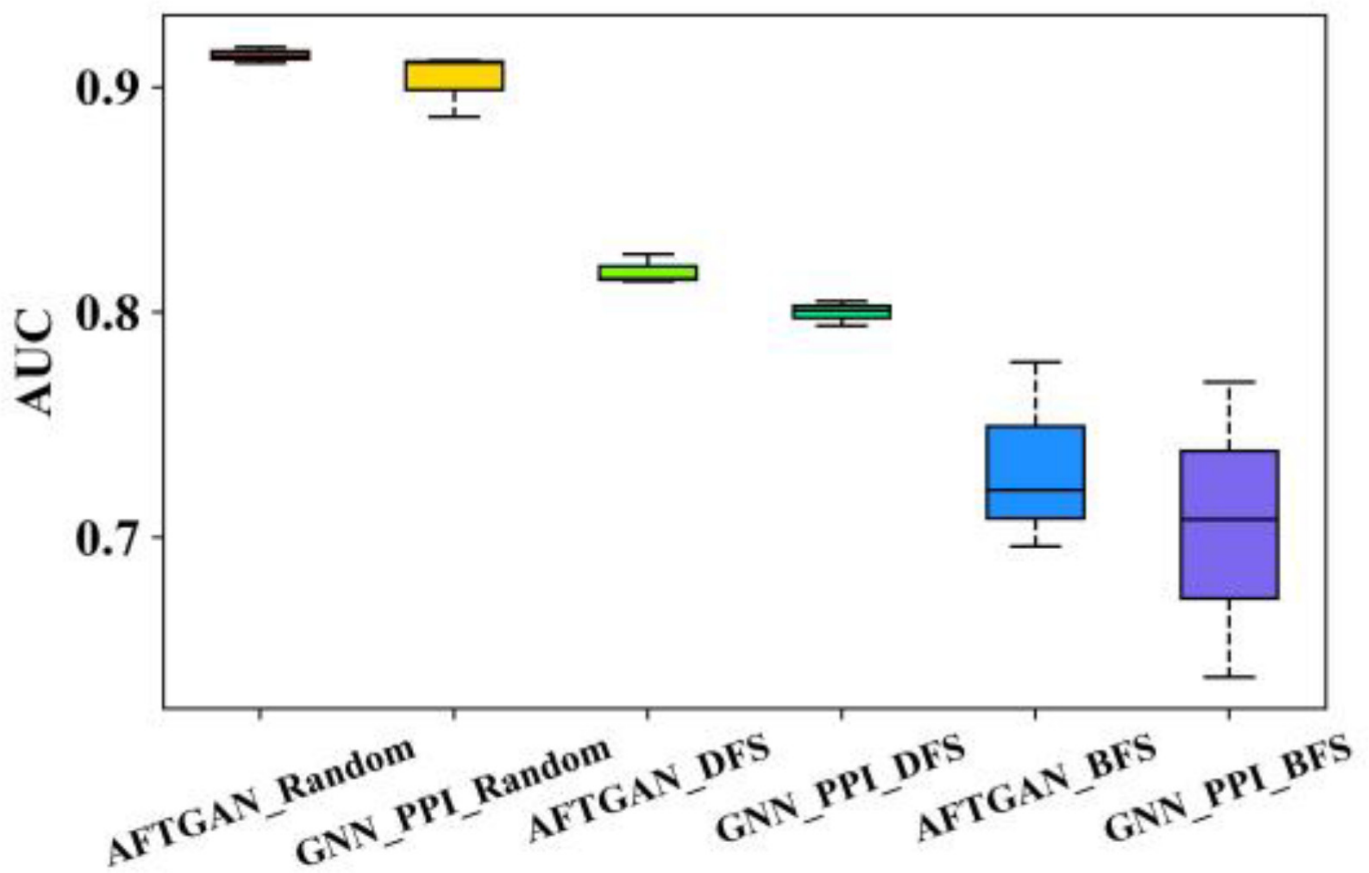
AUC results for three experiments of AFTGAN and GNN_PPI on SHS148K

To further illustrate the performance advantage of the proposed model, we compared the proposed AFTGAN with the previous best-performing method GNN-PPI in detail. The PR curves of three experiments of the AFTGAN and GNN_PPI on the SHS27K and SHS148K datasets are shown in Figure 4 and Supplementary Figure S1. AUC results for three experiments of the AFTGAN and the GNN_PPI on the SHS27K and SHS148K datasets are shown in Figure 5 and Supplementary Figure S2. The detailed experimental results are listed in Supplementary Table S6. We found when the test set is randomly divided: the Micro-F1 and Hamming Loss of the AFTGAN on the SHS27K reach 0.867 and 0.087, respectively, which are 0.3% and 0.001 higher than the GNN_PPI method. The Micro-F1 and Hamming Loss of the AFTGAN on the SHS148K reach 0.920 and 0.052, respectively, which are 0.2% and 0.001 higher than the GNN_PPI method. It can be seen that both the proposed AFTGAN and the GNN_PPI method show good performance when the test set is randomly divided, and the AFTGAN is slightly better than the GNN_PPI. When the test set is divided by BFS: the Micro-F1 and Hamming Loss of the AFTGAN on the SHS27K reach 0.685 and 0.191, respectively, which are 6.5% and 0.03 higher than the GNN_PPI method. The Micro-F1 and Hamming Loss of the AFTGAN on the SHS148K reach 0.745 and 0.159, respectively, which are 0.6% and 0.005 higher than the GNN_PPI method. For the PR curves of two datasets, it can be seen that the performance of the proposed AFTGAN is better than that of the GNN_PPI method under the BFS partition mode. When the test set is divided by DFS: the Micro-F1 and Hamming Loss of the AFTGAN on SHS27K reach 0.711 and 0.185, respectively, 5.9% and 0.028 higher than the GNN_PPI method. The Micro-F1 and Hamming Loss of the AFTGAN on SHS148K reach 0.819 and 0.119, respectively, 3.9% and 0.018 higher than the GNN_PPI method. For the PR curves of two datasets, the performance of AFTGAN is better than that of GNN_PPI under the DFS partitioning. The above experiments suggest that when the test set is divided by BFS and DFS, the proposed AFTGAN has a larger performance advantage on small datasets. We conclude that since the graph convolution framework in our network framework adopts a type of the GNN in the spatial domain, the updated features of protein nodes are only related to the neighboring nodes and independent of the graph structure. The advantages of the proposed model are obvious when the graph network contains less information.

In order to verify the influence of the sequence identity for the PPI prediction, we produced a new sub-dataset from SHS27K2 with <20% sequence identity for the PPI prediction. Experimental results are presented in Supplementary Table S7. When the partitioning mode was random, although the proposed AFTGAN achieved the Micro-F1 and the Hamming Loss of 0.872 and 0.083, which are slightly lower than the results of GNN_PPI model, its test performance cannot truly reflect the effect of the prediction model compared to BFS and DFS modes (Lv *et al.*, 2021). When the partitioning mode was BFS, the proposed AFTGAN achieved the Micro-F1 and the Hamming Loss of 0.800 and 0.139, 2.5% and 0.033 higher than those of GNN_PPI. When the partitioning mode was DFS, the proposed model achieved the Micro-F1 and the Hamming Loss of 0.841 and 0.109, 3% and 0.022 higher than the results of GNN_PPI, respectively. These results show that the proposed AFTGAN is still advantageous for the PPI prediction when the dataset has the low sequence identity.

### 3.5 In-depth analysis

**Fig. 6.**
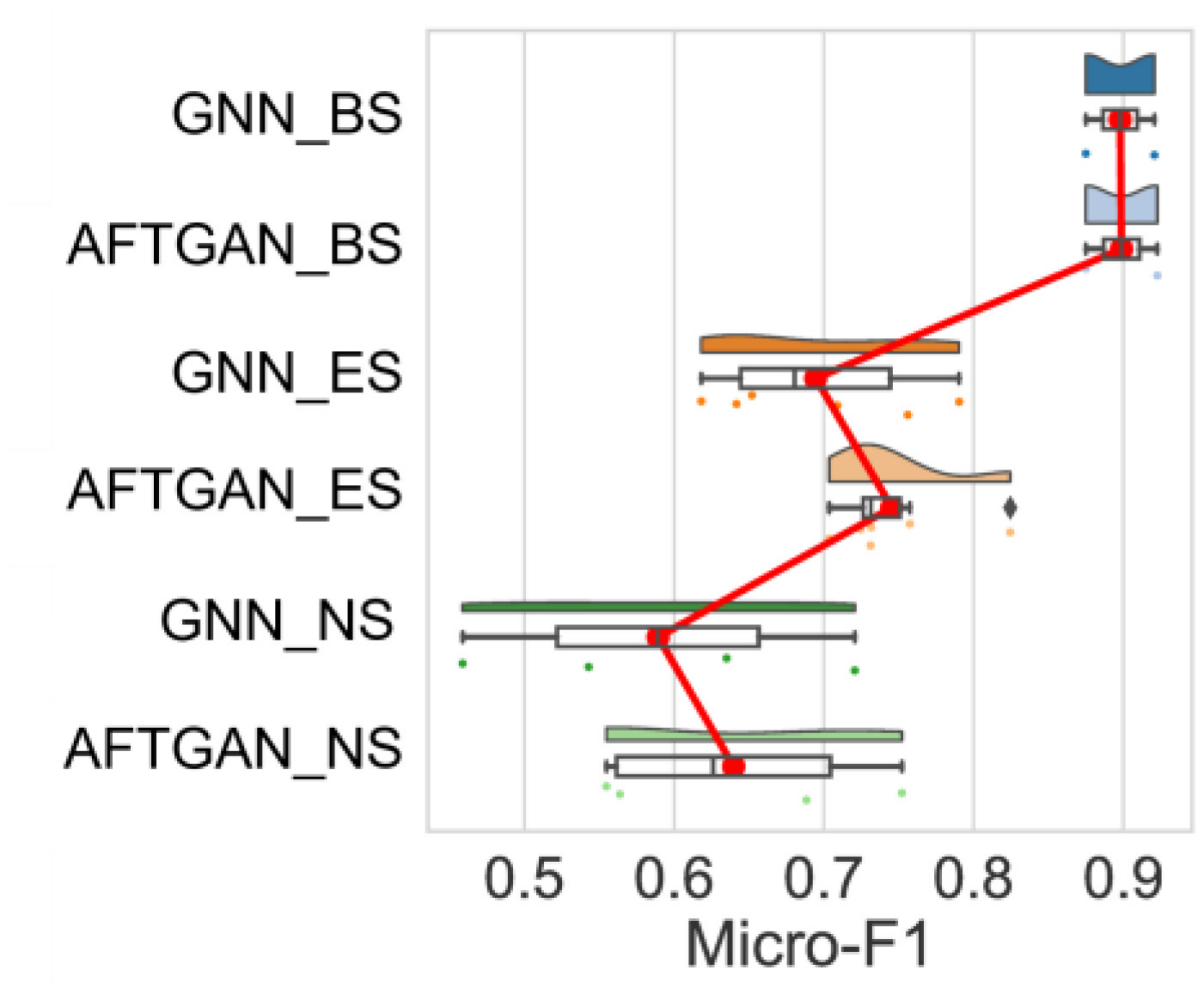
Micro-F1 comparison of AFTGAN and GNN_PPI in BS, ES and NS subsets

A more in-depth analysis was conducted on the interaction of two proteins about AFTGAN and GNN_PPI on the test set X (including SHS27K and SHS148K), as shown in Figure 6 (the red line represents the mean connecting line) and listed in Supplementary Table S8. Under the random partitioning mode, GNN_PPI and AFTGAN are not much different and show high performance in the X_BS subset. For the X_ES subset, the Micro-F1 of the AFTGAN is 8.3% higher than the GNN_PPI on the SHS27K test set and 0.2% higher than the GNN_PPI on the SHS148K test set. For SHS27K with the random division mode, the average number of elements in the X_ES subset by three times is 97, while the average number of elements in the X_ES subset by three times is 261 for SHS148K. Thus, the performance of the AFTGAN has a clear advantage over the performance of the GNN_PPI when the X_ES subset is small. With the increase of the number of the X_ES, the advantage weakens.

Under the BFS partition mode, Micro-F1 of the AFTGAN is 5.2% higher than the GNN_PPI on the SHS27K, and 2.3% higher than the GNN_PPI on the SHS148K for the X_ES subset. For the X_NS subset, Micro-F1 of the AFTGAN is 9.6% higher than the GNN_PPI on the SHS27K and 3.3% lower than the GNN_PPI on the SHS148K. The above result suggests that for the X_ES subset, the performance of the AFTGAN on both datasets is significantly improved compared with the GNN_PPI. For the X_NS subset, the performance of two methods on the SHS27K is not ideal, whereas the AFTGAN has a greater improvement than the GNN_PPI. On the SHS148K dataset, the performance of the AFTGAN and the GNN_PPI has been significantly improved, but the GNN_PPI has a more improvement. In brief, since the number of division elements of the X_ES is much larger than the number of division elements of the X_BS based on the BFS partition mode, the performance of the AFTGAN is still better than that of the GNN_PPI in the BFS partition mode.

Under the DFS partitioning mode, Micro-F1 of the AFTGAN is 11.3% higher than the GNN_PPI on the SHS27K test set and 3.5% higher than the GNN_PPI on the SHS148K for the X_ES subset. For the X_NS subset, Micro-F1 of the AFTGAN is 2.1% better than the GNN_PPI on the SHS27K, and 11.7% better than the GNN_PPI on the SHS148K. We found the performance of the AFTGAN has generally maintained a greater advantage than the GNN_PPI method based on the DFS mode.

From the analysis of three division subsets, the performance of two methods on the X_BS subset maintains high performance on both small datasets and large datasets, and there is little difference on the prediction performance. For the X_ES subset, the AFTGAN has the obvious advantage on small datasets, and the advantage gradually diminishes as the amount of data increases. For the X_NS subset, the performance of two methods is relatively low on small datasets. In comparison, the AFTGAN can maintain a stable performance under the BFS and DFS modes, while GNN_PPI has a significant performance drop based on the BFS mode. For the data with a large size, the performance of both methods is significantly improved, and the performance of the AFTGAN is more obvious based on the BFS mode.

### 3.6 Comparison of model generalization ability under different partition modes

**Fig. 7.**
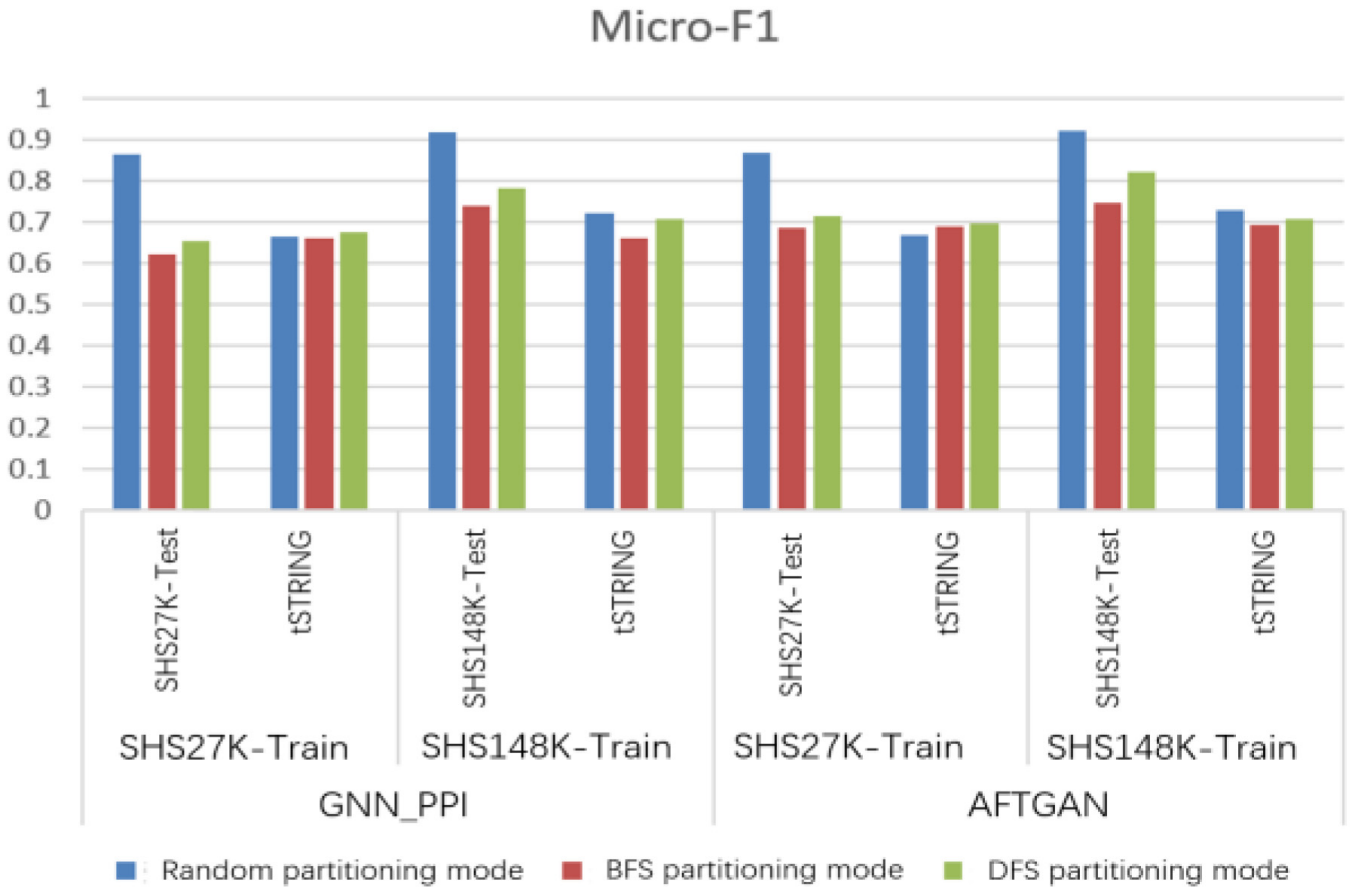
Micro-F1 comparison between AFTGAN and GNN_PPI under different partition modes, testing on the trainset-homologous test set and the unknown test set

To analyze the model generalization ability under different dataset partitioning modes, we chose the performance with the trained model on the large test set of *H.sapiens* in STRING (tSTRING) to compare the real generalization ability of the respective model. To be specific, the division results of the test set by different partitioning schemes are listed in Supplementary Table S9. The experimental results are shown in Figure 7 and listed in Supplementary Table S10. The results suggest that under the random partition mode, the performance on the unknown test set is significantly reduced by whether the GNN_PPI or AFTGAN method, suggesting that the random partition mode cannot truly reflect the generalization ability of the model again. However, the test results can truly reflect the performance of the prediction model based on the partition modes of DFS and BFS. For instance, during the experiment on the tSTRING test set, both methods use the SHS27K dataset as the training set, and when the test set is divided by BFS, the Micro-F1 of the AFTGAN is 2.9% better than the GNN_PPI. When the test set is divided by DFS, the Micro-F1 of the AFTGAN is 2.4% better than the GNN_PPI. When the SHS148K dataset is used as the training set, the experimental results suggest that AFTGAN also outperforms GNN_PPI no matter that the test set tSTRING is obtained by BFS or DFS.

### 3.7 Comparison of PPI graph network construction methods

**Fig. 8.**
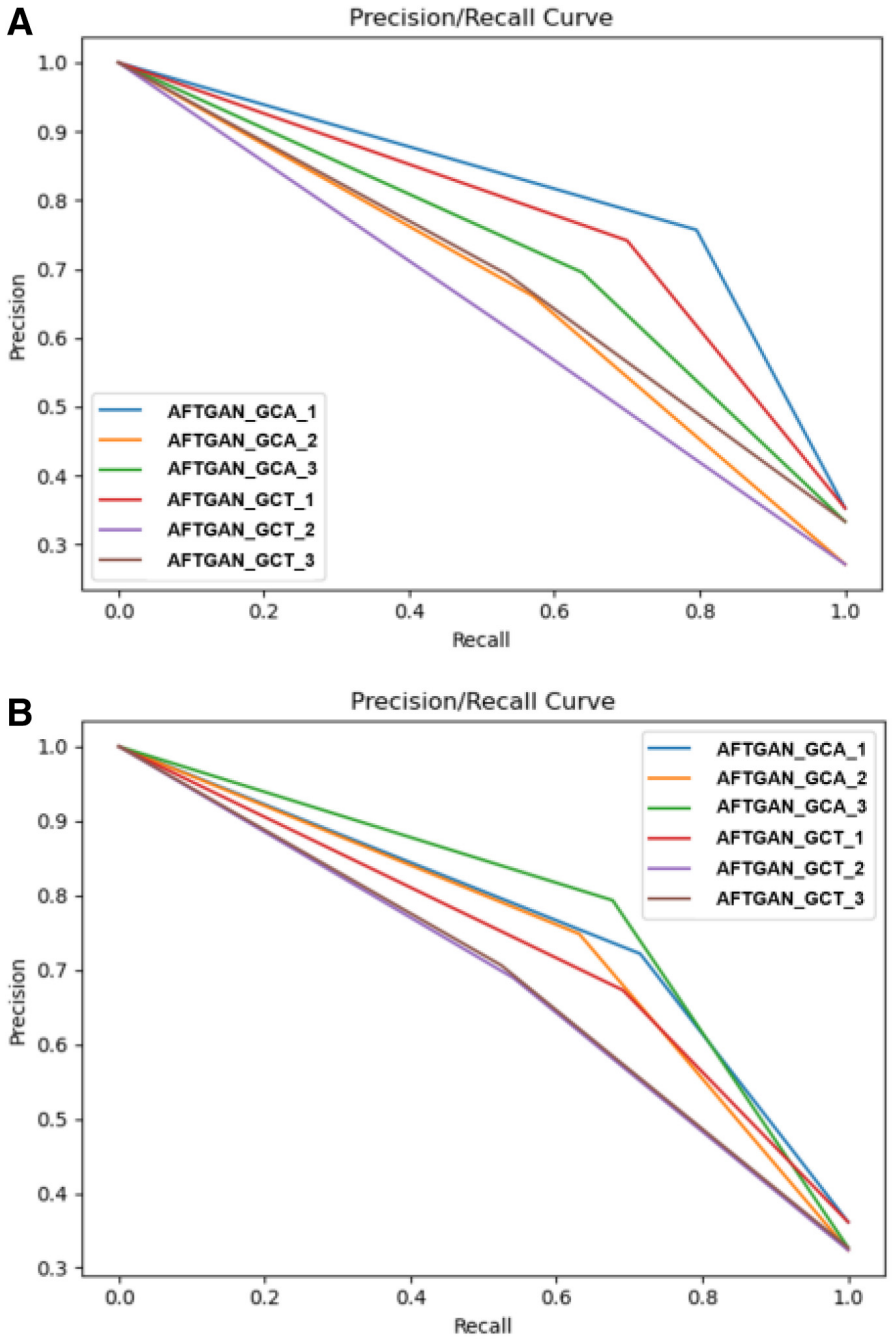
PR curves of three experiments for two PPI graph construction methods by AFTGAN and GNN_PPI on SHS27K. (**A**) Under the BFS partitioning. (**B**) Under the DFS partitioning

Lastly, the effect of PPI graph network construction methods in the AFTGAN and the GNN_PPI was analyzed. Two representation methods include the graph constructed by all data (GCA) and the graph constructed by the train set (GCT). The PPI prediction on the SHS27K dataset was compared, and the results are listed in Supplementary Table S11. Figure 8 presents the PR curves of three experiments for two PPI graph construction methods by the AFTGAN and the GNN_PPI. The above experimental results suggest that the performance of GCA exceeds that of GCT, whether it is AFTGAN or GNN_PPI. When the GCT construction by the AFTGAN and the GNN_PPI is compared, the Micro-F1 and Hamming Loss of the AFTGAN are improved by 3.5% and 0.02 compared with the GNN_PPI method under the BFS partitioning mode. In the DFS partition mode, the Micro-F1 and Hamming Loss of the AFTGAN are improved by 0.7% and 0.028 compared with those of the GNN_PPI method. The above results suggest that when predicting unknown proteins, the proposed AFTGAN has a greater predictive performance advantage when the unknown protein in the graph network is in a discrete state than when the unknown protein has an aggregated state. In addition, the two graph convolution-based GCT models still have much higher performance than the non-graph algorithms, which shows the superiority of graph convolution in few-shot learning for multi-label PPI prediction tasks. Moreover, for unknown proteins, we usually do not know their neighbors in advance. The effectiveness of GCT reveals that the trained model is robust to newly discovered proteins and their interactions.

## 4 Conclusion

In this study, a novel multi-type PPI prediction method (AFTGAN) is proposed based on attention free transformer and graph attention network. In the proposed AFTGAN, besides the embedding of amino acid co-occurrence similarity and the one-hot embedding composition of electrostatic and hydrophobic similarity between amino acids, ESM-1b embedding features are also added as the input features for the protein sequence feature. Moreover, proteins are used as the nodes, and seven types of protein interactions are used as the edges to construct seven PPI graphs as the link matrices. Subsequently, these graphs are input into the graph attention network. The neural network framework adopts the Transformer encoder containing the AFT module to extract protein sequence features. And the extracted protein sequence features are used as the PPI graph node features, which are input to the graph attention network with the constructed PPI graph to extract the relational features of protein pairs. Lastly, a FC serves as a classifier for the multi-label PPI prediction. Experimental results suggest that the proposed AFTGAN outperforms other popular methods using random, DFS and BFS partitioning schemes on the SHS27K and SHS148K datasets. To further illustrate the advantages of the AFTGAN in predicting unknown PPIs, the tSTRING dataset was selected as the test set for the experiment comparison. The results suggest that the proposed model also outperforms existing methods based on three partition modes.

Although the proposed model achieves the satisfactory prediction performance, there are still some contents for improvement in the future. The complete STRING was not used for training. We will learn the proposed model using STRING as the training set since more data information can enhance the performance of the model. For the feature input, the output of the pre-trained model of ESM-1b was adopted as one of protein codes. Since the length of the input sequence for ESM-1b was limited to 1024, thus resulting in a certain loss of protein sequence information. One of our future works will focus on how to extend the ESM-1b performance and add some new protein PPI features (e.g. the quality score) to enhance the model performance. In this study, the network framework combining AFT (a variant of the Transformer encoder) and GAT (a variant of the GNN) was used for the multi-type PPI prediction. In future research, we will also represent some new attention networks and their variants to further optimize the experimental results.

## Supplementary Material

btad052_Supplementary_Data
