# Supplementary material for: AFTGAN: prediction of multi-type PPI based on attention free transformer and graph attention network
Source: Bioinformatics. 2023 Jan 24;39(2):btad052. doi: 10.1093/bioinformatics/btad052 (PMC9897180; doi:10.1093/bioinformatics/btad052)
Supplement: btad052_Supplementary_Data [file btad052_supplementary_data.docx]

**Supplementary Materials**

**AFTGAN: Prediction of multi-type PPI based on attention free transformer and graph attention network**

#### Supplementary Figures


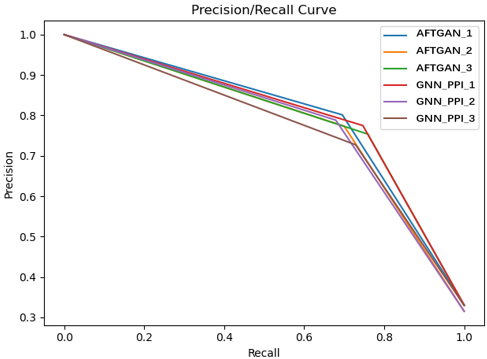

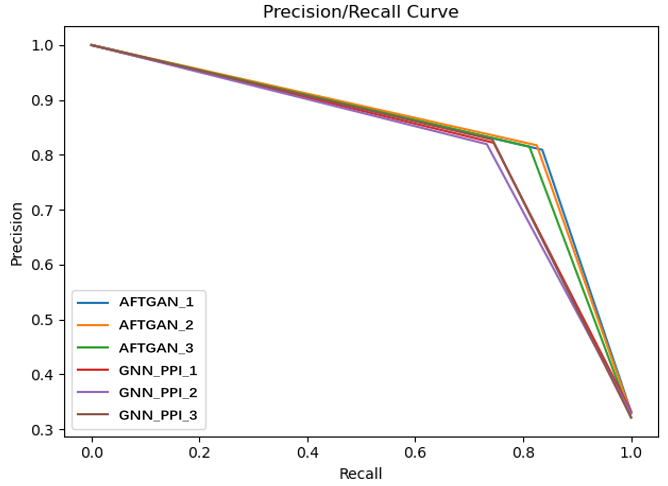


(A) Under the BFS partitioning (B) Under the DFS partitioning

**Figure S1**. PR curves of three experiments by AFTGAN and GNN_PPI on SHS148K.


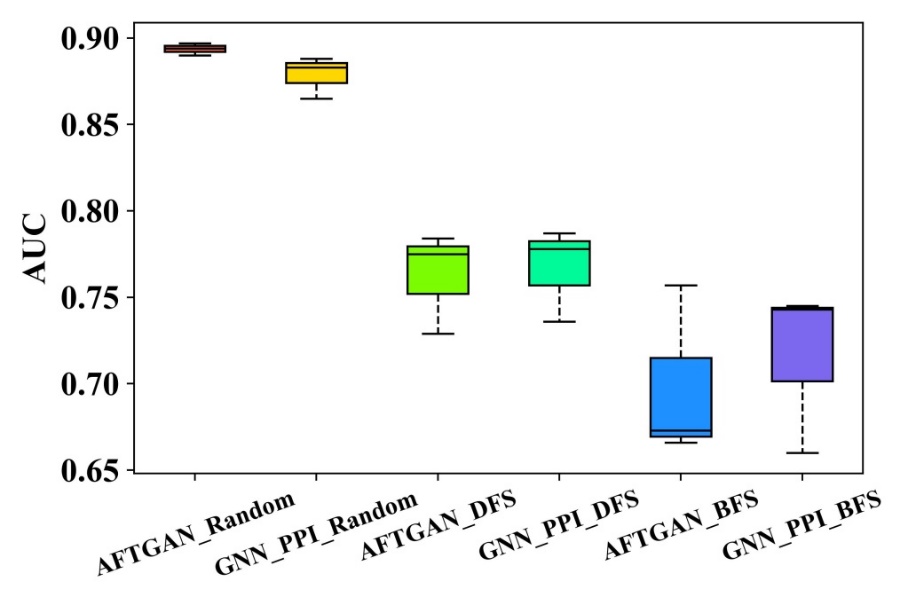


**Figure S2.** AUC results for three experiments of AFTGAN and GNN_PPI on SHS27K.

#### Supplementary Tables

**Table S1**. The classification of amino acids.

| No. | Dipole scale | Volume scale | Class |
| --- | --- | --- | --- |
| 1 | - | - | A, G, V |
| 2 | - | + | I, L, F, P |
| 3 | + | + | Y, M, T, S |
| 4 | ++ | + | H, N, E, W |
| 5 | +++ | + | R, K |
| 6 | +’+’+’ | + | D, E |
| 7 | +” | + | C |

Dipole scale: -, Dipole<1.0; +, 1.0<Dipole<2.0; ++, 2.0<Dipole<3.0; +++, Dipole>3.0; +'+'+', Dipole>3.0. Since Cystine (Cys) can form a disulfide bond, that is different from the third type of amino acids and is divided into the seventh type. Volume scale: -, Volume<50; +, Volume>50.

**Table S2**. Division results of the test set SHS27K by different partitioning schemes.

| Partition  scheme | No. | Vision node | Invisible node | X__BS_ | X__ES_ | X__NS_ |
| --- | --- | --- | --- | --- | --- | --- |
| Random | 1 | 1599 | 91 | 1428 | 93 | 4 |
|  | 2 | 1593 | 97 | 1424 | 98 | 3 |
|  | 3 | 1591 | 99 | 1417 | 101 | 7 |
| BFS | 1 | 1563 | 127 | 0 | 1211 | 319 |
|  | 2 | 1550 | 140 | 0 | 1310 | 280 |
|  | 3 | 1537 | 153 | 0 | 1173 | 357 |
| DFS | 1 | 1618 | 72 | 0 | 1388 | 167 |
|  | 2 | 1560 | 130 | 0 | 1321 | 221 |
|  | 3 | 1570 | 120 | 0 | 1301 | 265 |

**Table S3**. Effect verification of different input features on the SHS27K dataset.

| Method | Partition  Scheme | Precision | Recall | Micro-F1 | Hamming Loss |
| --- | --- | --- | --- | --- | --- |
| AFTGAN | Random | 0.862 | 0.871 | 0.867 | 0.087 |
|  | BFS | 0.705 | 0.668 | 0.685 | 0.191 |
|  | DFS | 0.755 | 0.674 | 0.711 | 0.185 |
| Without ESM-1b | Random | 0.831 | 0.798 | 0.814 | 0.117 |
|  | BFS | 0.658 | 0.609 | 0.631 | 0.225 |
|  | DFS | 0.593 | 0.668 | 0.629 | 0.285 |
| ESM-1V | Random | 0.823 | 0.807 | 0.815 | 0.118 |
|  | BFS | 0.693 | 0.630 | 0.660 | 0.206 |
|  | DFS | 0.647 | 0.663 | 0.655 | 0.253 |

**Table S4**. Verification of different modules on the SHS27K dataset.

| Method | Partition  scheme | Precision | Recall | Micro-F1 | Hamming Loss |
| --- | --- | --- | --- | --- | --- |
| AFTGAN | Random | 0.862 | 0.871 | 0.867 | 0.087 |
|  | BFS | 0.705 | 0.668 | 0.685 | 0.191 |
|  | DFS | 0.755 | 0.674 | 0.711 | 0.185 |
| Without AFT module | Random | 0.811 | 0.800 | 0.805 | 0.125 |
|  | BFS | 0.683 | 0.637 | 0.659 | 0.210 |
|  | DFS | 0.649 | 0.682 | 0.665 | 0.248 |
| Bidirectional RNN | Random | 0.759 | 0.785 | 0.772 | 0.150 |
|  | BFS | 0.652 | 0.638 | 0.643 | 0.224 |
|  | DFS | 0.696 | 0.720 | 0.708 | 0.202 |
| Bidirectional LSTM | Random | 0.682 | 0.731 | 0.704 | 0.200 |
|  | BFS | 0.616 | 0.578 | 0.594 | 0.248 |
|  | DFS | 0.648 | 0.667 | 0.657 | 0.237 |
| GIN module | Random | 0.861 | 0.869 | 0.864 | 0.089 |
|  | BFS | 0.699 | 0.600 | 0.645 | 0.209 |
|  | DFS | 0.730 | 0.685 | 0.704 | 0.198 |

**Table S5**. Division results of the test set SHS148K by different partitioning schemes.

| Partition scheme | No. | Vision node | Invisible node | X__BS_ | X__ES_ | X__NS_ |
| --- | --- | --- | --- | --- | --- | --- |
| Random | 1 | 4960 | 229 | 8646 | 250 | 2 |
|  | 2 | 4967 | 222 | 8640 | 256 | 2 |
|  | 3 | 4946 | 243 | 8619 | 276 | 3 |
| BFS | 1 | 4779 | 410 | 0 | 6767 | 2210 |
|  | 2 | 4643 | 546 | 0 | 7701 | 1263 |
|  | 3 | 4671 | 518 | 0 | 5353 | 3553 |
| DFS | 1 | 4919 | 270 | 0 | 8242 | 705 |
|  | 2 | 4897 | 292 | 0 | 8141 | 792 |
|  | 3 | 4904 | 285 | 0 | 8226 | 722 |

**Table S6**. Performance comparison of AFTGAN and GNN-PPI on SHS27K and SHS148K.

| Method | Dataset | Partition scheme | Precision | Recall | Micro-F1 | Hamming  Loss |
| --- | --- | --- | --- | --- | --- | --- |
| AFTGAN | SHS27K | Random | 0.862 | 0.871 | 0.867 | 0.087 |
|  |  | BFS | 0.705 | 0.668 | 0.685 | 0.191 |
|  |  | DFS | 0.755 | 0.674 | 0.711 | 0.185 |
|  | SHS148K | Random | 0.929 | 0.910 | 0.920 | 0.052 |
|  |  | BFS | 0.776 | 0.718 | 0.745 | 0.159 |
|  |  | DFS | 0.814 | 0.824 | 0.819 | 0.119 |
| GNN-PPI | SHS27K | Random | 0.871 | 0.857 | 0.864 | 0.086 |
|  |  | BFS | 0.677 | 0.572 | 0.620 | 0.221 |
|  |  | DFS | 0.678 | 0.629 | 0.652 | 0.213 |
|  | SHS148K | Random | 0.925 | 0.911 | 0.918 | 0.053 |
|  |  | BFS | 0.764 | 0.718 | 0.739 | 0.164 |
|  |  | DFS | 0.823 | 0.740 | 0.780 | 0.137 |

**Table S7**. Performance comparison of AFTGAN and GNN-PPI on the sub-dataset from SHS27K with less than 20% sequence identity.

| Method | Dataset | Partition scheme | Precision | Recall | Micro-F1 | Hamming Loss |
| --- | --- | --- | --- | --- | --- | --- |
| AFTGAN | Sub-dataset from SHS27K | Random | 0.871 | 0.872 | 0.872 | 0.083 |
|  |  | BFS | 0.812 | 0.789 | 0.800 | 0.139 |
|  |  | DFS | 0.812 | 0.872 | 0.841 | 0.109 |
| GNN-PPI | Sub-dataset from SHS27K | Random | 0.886 | 0.880 | 0.883 | 0.075 |
|  |  | BFS | 0.781 | 0.712 | 0.775 | 0.172 |
|  |  | DFS | 0.776 | 0.850 | 0.811 | 0.131 |

**Table S8**. Micro-F1 comparison of AFTGAN and GNN_PPI in BS, ES and NS subsets.

| Dataset | Partition scheme | X__BS_ | | X__ES_ | | X__NS_ | | $X_{\mathrm{AVG}}$ | |
| --- | --- | --- | --- | --- | --- | --- | --- | --- | --- |
|  |  | GNN-PPI | AFT  GAN | GNN-PPI | AFT  GAN | GNN-PPI | AFT  GAN | GNN-PPI | AFT  GAN |
| SHS27K | Random | 0.875 | 0.875 | 0.642 | 0.725 | - | - | 0.864 | 0.867 |
|  | BFS | - | - | 0.652 | 0.704 | 0.459 | 0.555 | 0.620 | 0.685 |
|  | DFS | - | - | 0.618 | 0.731 | 0.543 | 0.564 | 0.652 | 0.711 |
| SHS148K | Random | 0.921 | 0.923 | 0.756 | 0.758 | - | - | 0.918 | 0.920 |
|  | BFS | - | - | 0.709 | 0.732 | 0.721 | 0.688 | 0.739 | 0.745 |
|  | DFS | - | - | 0.790 | 0.825 | 0.635 | 0.752 | 0.780 | 0.819 |

**Table S9**. Division results of Homo sapiens test set in STRING (tSTRING) by different partitioning schemes.

| Partition mode | Vision node | Invisible node | X__BS_ | X__ES_ | X__NS_ |
| --- | --- | --- | --- | --- | --- |
| Random | 15073 | 262 | 118375 | 305 | 0 |
| BFS | 14287 | 1048 | 0 | 85913 | 32795 |
| DFS | 14564 | 771 | 0 | 111695 | 7005 |

**Table S10**. Micro-F1 comparison of AFTGAN and GNN_PPI under different partitioning modes, testing on the trainset-homologous test set and unknown test set.

| Method | Training set | Test set | Partition mode | | |
| --- | --- | --- | --- | --- | --- |
|  |  |  | Random | BFS | DFS |
| GNN_PPI | SHS27K-Train | SHS27K-Test | 0.864 | 0.620 | 0.652 |
|  |  | tSTRING | 0.663 | 0.659 | 0.673 |
|  | SHS148K-Train | SHS148K-Test | 0.918 | 0.739 | 0.780 |
|  |  | tSTRING | 0.720 | 0.660 | 0.706 |
| AFTGAN | SHS27K-Train | SHS27K-Test | 0.867 | 0.685 | 0.711 |
|  |  | tSTRING | 0.666 | 0.688 | 0.697 |
|  | SHS148K-Train | SHS148K-Test | 0.920 | 0.745 | 0.819 |
|  |  | tSTRING | 0.726 | 0.690 | 0.707 |

**Table S11.** Comparison of Micro-F1 on SHS27K between GNN-PPI and AFTGAN using different PPI Graph construction methods.

| Method | Graph | Partition scheme | Precision | Recall | Micro-F1 | Hamming Loss |
| --- | --- | --- | --- | --- | --- | --- |
| AFTGAN | GCA | BFS | 0.705 | 0.668 | 0.685 | 0.191 |
|  |  | DFS | 0.755 | 0.674 | 0.711 | 0.185 |
|  | GCT | BFS | 0.676 | 0.599 | 0.634 | 0.216 |
|  |  | DFS | 0.689 | 0.585 | 0.630 | 0.229 |
| GNN_PPI | GCA | BFS | 0.572 | 0.677 | 0.620 | 0.221 |
|  |  | DFS | 0.629 | 0.678 | 0.652 | 0.213 |
|  | GCT | BFS | 0.652 | 0.556 | 0.599 | 0.236 |
|  |  | DFS | 0.664 | 0.586 | 0.623 | 0.257 |
